# Interface Engineering via Regulating Electrolyte for High‐Voltage Layered Oxide Cathodes‐Based Li‐Ion Batteries

**DOI:** 10.1002/advs.202206714

**Published:** 2023-02-19

**Authors:** Fangyuan Cheng, Jia Xu, Peng Wei, Zexiao Cheng, Mengyi Liao, Shixiong Sun, Yue Xu, Qing Li, Chun Fang, Yaqing Lin, Jiantao Han, Yunhui Huang

**Affiliations:** ^1^ State Key Laboratory of Material Processing and Die & Mould Technology School of Materials Science and Engineering Huazhong University of Science and Technology Wuhan Hubei 430074 P. R. China

**Keywords:** graphite anodes, high‐voltage electrolytes, interface engineering, Li‐rich/Ni‐rich cathodes, lithium‐ion batteries

## Abstract

Li‐rich and Ni‐rich layered oxides as next‐generation high‐energy cathodes for lithium‐ion batteries (LIBs) possess the catalytic surface, which leads to intensive interfacial reactions, transition metal ion dissolution, gas generation, and ultimately hinders their applications at 4.7 V. Here, robust inorganic/organic/inorganic‐rich architecture cathode‐electrolyte interphase (CEI) and inorganic/organic‐rich architecture anode‐electrolyte interphase (AEI) with F‐, B‐, and P‐rich inorganic components through modulating the frontier molecular orbital energy levels of lithium salts are constructed. A ternary fluorinated lithium salts electrolyte (TLE) is formulated by mixing 0.5 m lithium difluoro(oxalato)borate, 0.2 m lithium difluorophosphate with 0.3 m lithium hexafluorophosphate. The obtained robust interphase effectively suppresses the adverse electrolyte oxidation and transition metal dissolution, significantly reduces the chemical attacks to AEI. Li‐rich Li_1.2_Mn_0.58_Ni_0.08_Co_0.14_O_2_ and Ni‐rich LiNi_0.8_Co_0.1_Mn_0.1_O_2_ in TLE exhibit high‐capacity retention of 83.3% after 200 cycles and 83.3% after 1000 cycles under 4.7 V, respectively. Moreover, TLE also shows excellent performances at 45 °C, demonstrating this inorganic rich interface successfully inhibits the more aggressive interface chemistry at high voltage and high temperature. This work suggests that the composition and structure of the electrode interface can be regulated by modulating the frontier molecular orbital energy levels of electrolyte components, so as to ensure the required performance of LIBs.

## Graphical abstract

Excellent high voltage performance under 4.7 V for 1000 cycles with LiNi_0.8_Co_0.1_Mn_0.1_O_2_ cathode and 200 cycles with Li_1.2_Mn_0.58_Ni_0.08_Co_0.14_O_2_ cathode by optimizing electrolyte to form a robust inorganic/organic/inorganic‐rich architecture cathode‐electrolyte interphase and inorganic/organic‐rich architecture anode‐electrolyte interphase (AEI), which suppresses the adverse electrolyte oxidation and transition metal dissolution, significantly reduces the chemical attacks to AEI.

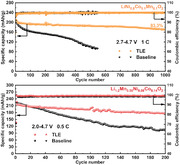

## 1 Introduction

The expanding application of lithium‐ion batteries (LIBs) in electric vehicles and large‐scale energy storage promotes the rapid development of low cost and high‐energy‐density cathodes.^[^ ^1^ ^]^ Reducing the use of expensive elements (such as cobalt) is essential to decrease the cost of cathode.^[^ ^1^, ^2^ ^]^ To increase the energy density, it is required to either enhance the specific capacity or the upper cut‐off voltage of the cathode. Li‐rich layered oxides (LRLOs) and Ni‐rich layered oxides (NRLOs) have obtained great attention due to their relatively low costs and high specific capacities to 250 and 220 mAh g^−1^ when charged to 4.7 V (vs Li^+^/Li), respectively.^[^ ^3^ ^]^ However, they suffer from rapid capacity fading, sluggish kinetics, and severe voltage decay, which are largely associated with the irreversible surface structural transitions and dissolution of transition metal ions.^[^ ^4^ ^]^ At high charge voltage beyond 4.5 V (vs Li^+^/Li), the O^2−^ would participate in the oxidation reaction, resulting in irreversible surface oxygen loss of LRLOs and NRLOs.^[^ ^5^ ^]^ Furthermore, the transition metal ions would migrate to the Li site on high state of charge, triggering the formation of the spinel and rock salt phase on the surface, and generating cracks and pores.^[^ ^4^, ^5^ ^]^ Meanwhile, the oxidation and decomposition of the commercial ethylene carbonate (EC)‐based electrolytes accelerates when the charging voltage exceeds 4.3 V, causing severe side reactions at cathode‐electrolyte interface (CEI) and the dissolution of transition metals.^[^ ^6^ ^]^ All of the above reactions will cause severe structure degradations of LRLOs and NRLOs and reduce the cycle life and average discharge voltage of LIBs. Besides, the transition metal ions dissolved from the cathodes are reduced and deposited on the anode, which destroys the anode‐electrolyte interphase (AEI) and eventually causes the co‐intercalation of Li^+^ and solvent in graphite, resulting in the exfoliation of the graphite.^[7]^

The commonly used modification methods to improve the stability of cathodes include surface coating, elements doping, and electrolyte formulation. Among them, the electrolyte modification is a simpler method, which can effectively improve the CEI stability and prevent the surface structure degradation under high voltage. Although conventional carbonate and LiPF_6_‐based electrolytes have been widely applied due to their high Li^+^ conductivity and passivation effect on Al current collector,^[^ ^8^ ^]^ they have narrow electrochemical stability windows (below 4.3 V vs Li/Li^+^) and are highly water sensitive.^[^ ^9^ ^]^ Furthermore, the high content of PF_6_^−^ in the electrolyte would significantly reduce the oxidation onset potential of EC, and the EC in the solvation structure will undergo H transfer when it loses electrons, and react with the surrounding PF_6_^−^ to generate hydrogen fluoride (HF), which significantly deteriorate the stability of electrolyte at high voltage.^[^ ^10^ ^]^ Usually, two strategies were used to widen the electrochemical stability window, including extending the intrinsic electrolyte stability window thermodynamically, or constructing stable AEI and CEI to inhibit the electrolyte decomposition from the perspective of dynamics.^[^ ^6^ ^]^ Among them, kinetically widening the electrochemical stability window by manipulating the interface is low cost and easy to commercial. The appropriate molecules with lower LUMO (lowest unoccupied molecular orbital) and higher HOMO (highest occupied molecular orbital) can be decomposed preferentially and construct stable and compact AEI and CEI, respectively, eventually expand the electrochemical stability window. It is worth mentioning that both lithium difluoro(oxalate)borate (LiDFOB) and lithium difluorophosphate (LiDFP) have lower LUMO and higher HOMO than LiPF_6_ and EC,^[^ ^11^ ^]^ establishing robust CEI and SEI.^[^ ^11^, ^12^ ^]^ Moreover, PO_2_F_2_^−^ dissociated from LiDFP is the decomposition intermediate of PF_6_^−^, which can efficiently suppress the decomposition of PF_6_^−^.^[^ ^12^ ^]^ The (oxalate)borate in LiDFOB could suppress the decompositions of carbonate electrolytes by complexing with the anions and scavenging HF.^[^ ^6^, ^7^ ^]^ And LiDFOB can also improve cycle performance at high and low temperature.^[^ ^13^ ^]^ Therefore, using LiDFP and LiDFOB to partially replace LiPF_6_ can improve the electrolyte stability and construct robust CEI and AEI to improve the cycle stability at high voltage.

In this work, to ensure a stable electrochemical window at 4.7 V and construct a high‐voltage resistant interface, LiDFOB and LiDFP with lower LUMO and higher HOMO than LiPF_6_ and EC were chosen to replace part of LiPF_6_ to formulate into ternary fluorinated lithium salts electrolyte (TLE) by mixing 0.5 m LiDFOB, 0.2 m LiDFP, and 0.3 m LiPF_6_ in EC/diethyl carbonate (DEC, volume ratio 1:1). LiDFP and LiDFOB significantly increase the content of inorganic components in the CEI and AEI. These F‐, B‐, and P‐rich inorganic components can not only improve interface stability and reduce the damage of transition metal dissolution to the interface film on anode, but also beneficial to the high temperature performance of the cell with high‐voltage LRLOs and NRLOs cathode. At a high cut‐off voltage of 4.7 V, the Li/Li_1.2_Mn_0.58_Ni_0.08_Co_0.14_O_2_ half‐cell exhibits a capacity retention of 83.3% after 200 cycles and Li/LiNi_0.8_Co_0.1_Mn_0.1_O_2_ half‐cell reaches a high capacity retention of 83.3% after 1000 cycles. Moreover, the graphite/Li_1.2_Mn_0.58_Ni_0.08_Co_0.14_O_2_ full‐cell exhibits high‐capacity retention of 85.1% after 200 cycles at 2.0–4.65 V, and graphite/LiNi_0.8_Co_0.1_Mn_0.1_O_2_ full‐cell shows 81% capacity after 600 cycles at 2.7–4.5 V. More significantly, TLE also guarantees the excellent performance of the battery at 45 °C, indicating this inorganic rich interface is also durable under high voltage and high temperature. This work enlightens us that the properties of the electrode interface film can be regulated to improve the battery performances by modulating the frontier molecular orbitals of each component in the electrolyte, which is easy to realize in the commercialization application.

## 2 Results and Discussion

### 2.1 The Electrochemical Performance of LRLOs and Graphite

**Figure 1:**
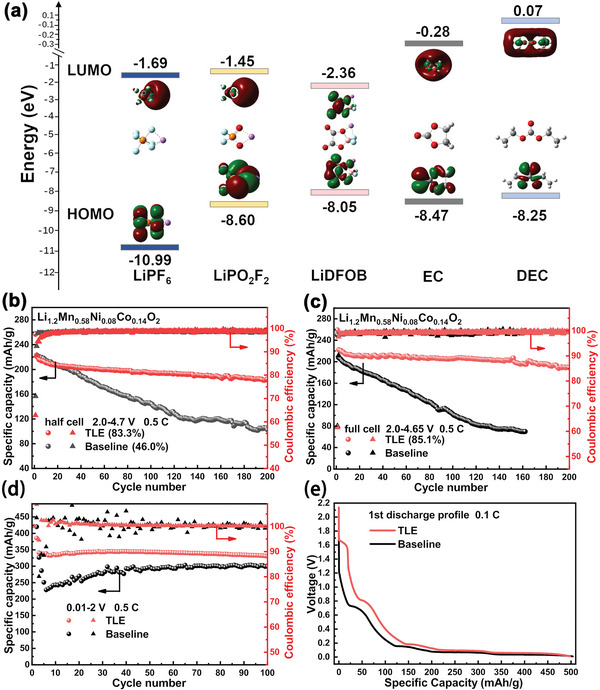
a) The frontier molecular orbital energy levels of each component in the electrolyte, cycle performance of b) Li/Li_1.2_Mn_0.58_Ni_0.08_Co_0.14_O_2_ half‐cells, c) graphite/Li_1.2_Mn_0.58_Ni_0.08_Co_0.14_O_2_ full‐cells, and d) Li/graphite half‐cells with baseline and TLE as electrolyte, e) the first discharge profiles of graphite at 0.1 C.

The high‐voltage compatibility depends on the ability of electrolyte to resist electrochemical oxidative decomposition. Thermodynamically, a high‐voltage stable electrolyte means that all the components of electrolyte (solvent, lithium salts, and additives) should be simultaneously stable enough to have a lower HOMO energy relative to the cathode.^[^ ^14^ ^]^ However, due to the strong oxidation of the cathode materials during lithium deintercalation process, the electrolyte will be oxidized by the delithiated cathode and lead to complicated interfacial parasitic reactions. To suppress the side reactions that are fatal to the cycle stability, a chemical passivation interphase should be constructed at the cathode‐electrolyte interface, namely, CEI. Similarly, due to the higher energy levels of the LUMO of the lithium graphite anode, almost all solvents in the traditional carbonate electrolyte will be reduced on the anode. To prevent side reactions at the AEI, an AEI passivation layer should also be required. According to the theoretical calculation (**Figure** **1**a), the HOMO of EC and DEC was lower than that of LiDFOB and higher than that of LiPF_6_ and LiDFP, which means that solvent should participate in the CEI reaction after LiDFOB decomposition and before the decomposition of LiPF_6_ and LiDFP. Therefore, it tends to form an inorganic–organic–inorganic CEI layer from inside to outside on the cathode surface. As for the anode, the LUMO energy levels of LiPF_6_, LiDFP, and LiDFOB are all lower than that of EC and DEC, indicating that these three lithium salts will decompose prior to the solvent. As a result, an inside inorganic‐rich and outside organic‐rich AEI layer will be constructed on the anode surface. The Raman spectra of the electrolytes were tested as shown in Figure S1 in the Supporting Information to elucidate the solvation structures. Compared to the pure solvents (EC:DEC = 1:1, by volume), baseline (1 m LiPF_6_/EC:DEC = 1:1, by volume) and TLE (0.5 m LiDFOB + 0.2 m LiDFP + 0.3 m LiPF_6_/EC:DEC = 1:1, by volume) exhibit two additional peaks assigned to the free anion and Li^+^‐solvent coordination cluster, respectively. The difference is that the free anion in TLE is much smaller than baseline, and the Li^+^‐solvent peak is also significantly weaker than baseline, which indicates that the addition of LiDFOB and LiDFP reduces the free anions, makes more participation in the solvation structure, thus reducing the Li^+^‐solvent peak, which is beneficial to the formation of inorganic‐rich interface film.^[^ ^15^ ^]^

Figure S2a,b in the Supporting Information shows the cyclic voltammetry (CV) curves of baseline and TLE electrolyte within 2.0–6.0 V (vs Li/Li^+^) on Li/stainless steel cells. It can be seen from Figure S2a in the Supporting Information that the onset oxidation potential of the first circle in baseline is lower than in TLE, demonstrating LiDFP and LiDFOB can suppress the decomposition of electrolyte below 4.5 V. The oxidation peak of TLE is stronger, which may be attributed to the oxidative decomposition of LiDFOB and LiDFP and the formation of CEI. However, CV curve of the second cycle varies greatly between baseline and TLE (Figure S2b, Supporting Information). It is clear that there is no obvious oxidation peak for TLE, indicating that the CEI formed in TLE in the first cycle is complete and robust. While the baseline electrolyte still continuously decomposes in the following cycles due to the unstable interface and the formation of HF in the first cycle.^[^ ^9^ ^]^ The obvious difference is that the cell with TLE has two reduction peaks in the voltage range of 2.0–3.0 V, which might derive from the decomposition of LiDFOB and LiDFP. The above comparison shows that CEI and AEI can be formed in both baseline electrolyte and TLE, but the CEI formed in TLE is more robust and can improve the electrode–electrolyte interface stability during the subsequent charge and discharge process.

Figure 1b and Figures S3 and S4 in the Supporting Information show the cycle performance of the Li/Li_1.2_Mn_0.58_Ni_0.08_Co_0.14_O_2_ half‐cells with baseline and TLE as electrolyte in the voltage range of 2.0–4.7 V at 0.5 C (1 C = 250 mAh g^−1^). The retention of discharge capacity and average discharge voltage of the TLE cell achieves 83.3% and 93.0% over 200 cycles, while those of the baseline cell is only 46.0% and 82.0%, respectively. As shown in Figure 1c, the discharge capacity of graphite/Li_1.2_Mn_0.58_Ni_0.08_Co_0.14_O_2_ full‐cell with TLE is 189 mA h g^−1^ after 200 cycles, corresponding to a capacity retention of 85.1% higher than that of the half‐cell. As a comparison, the baseline full‐cell suffered from a more severe capacity fading, with a capacity of 70 mA h g^−1^ after 160 cycles. Compared to half‐cells, the increased capacity retention of TLE full‐cell and decreased capacity retention of baseline full‐cell indicate that there is a crossover effect between cathode and anode in baseline cell, but it is alleviated in the TLE cell.^[^ ^16^ ^]^ And as shown in Figure 1d, the Li/graphite cell with TLE also shows higher capacity and more stable cycle performance than baseline cell especially at initial cycles. This is due the formation of robust AEI at around 1.65 V in the first discharge process in TLE cell, as shown in Figure 1e, which mainly derives from the decomposition of LiDFOB and LiDFP on the graphite–electrolyte interface. Figure S5 in the Supporting Information shows the more excellent cycle performance of Li/Li_1.2_Mn_0.58_Ni_0.08_Co_0.14_O_2_ pouch cell in TLE cell than baseline cell. The pouch cell with TLE delivers higher capacity retention of 85.6% after 100 cycles and high Coulombic efficiency of 99.7%. The significant improvements show that the 4.7 V LRLOs cathode has the potential for commercial application by reasonably tailoring the electrolyte to construct stable interfaces at both cathode and anode.

### 2.2 Formation Mechanisms of CEI and AEI

**Figure 2:**
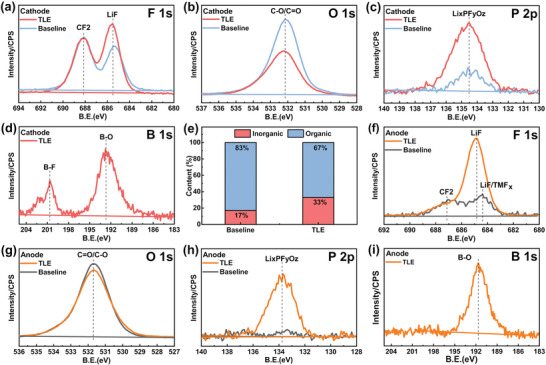
XPS spectra of CEI and AEI layer of graphite/Li_1.2_Mn_0.58_Ni_0.08_Co_0.14_O_2_ full‐cells with TLE and baseline as electrolyte after 10 cycles: a–d) CEI and f–i) AEI, e) the contents of organic and inorganic components in CEI after ten cycles.

In order to investigate the interface composition, the surface chemistry of the Li_1.2_Mn_0.58_Ni_0.08_Co_0.14_O_2_ cathode and graphite anode after ten cycles of the graphite/Li_1.2_Mn_0.58_Ni_0.08_Co_0.14_O_2_ full‐cells were characterized by X‐ray photoelectron spectroscopy (XPS) in **Figure** **2**. As shown in Figure 2a–d, the CEI formed in TLE is richer in inorganic components composed of LiF, Li*_x_* PO*_y_* F*_z_*, B*_x_* F*_y_*, and B*_x_* O*_y_*, while the CEI derived from baseline electrolyte contains higher content of organic compounds (C—O/C=O in O 1s spectrum), which is also confirmed by the relative percentage of organic and inorganic components in CEI obtained by XPS fitting (Figure S6, Supporting Information and Figure 2e). It is revealed that TLE promotes the formation of inorganic components rich in F, P, and B, while reducing the content of organic components. According to the energy levels of HOMO (Figure 1a), LiDFOB and LiDFP decompose simultaneously with the solvent molecules to increase the inorganic components in the interface on cathode surface, which is more beneficial to interface stability than the CEI rich in organic compounds, as well as inhibit the adverse reactions at the CEI.^[^ ^17^ ^]^ Figure 2f–i and Figure S7 in the Supporting Information show the XPS spectra of the graphite anodes cycled in TLE and baseline electrolytes. The F 1s, P 2p, and B 1s peak display that the AEI of TLE cell is dramatically rich in LiF, Li*_x_* PO*_y_* F*_z_*, and B*_x_* O*_y_*, while the AEI of baseline cell is mainly composed of organic compounds derived from solvent decomposition as shown in Figure S8 in the Supporting Information. More inorganic components can prevent the AEI from being catalytically decomposed by the transition metal dissolved from the cathode. Based on the calculation of the LUMO energy level in Figure 1a, the increase of inorganic components is caused by the lower LUMO energy level of LiDFOB and LiDFP, which will be decomposed preferentially to EC and DEC on the anode surface.

**Figure 3:**
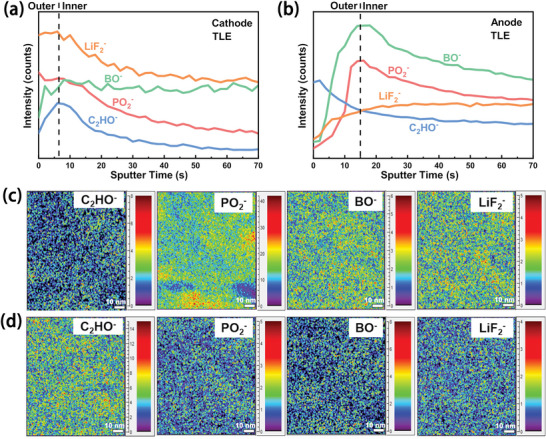
TOF‐SIMS depth profiles and surface mapping pictures of cycled cathode and anode of graphite/Li_1.2_Mn_0.58_Ni_0.08_Co_0.14_O_2_ full‐cells with TLE. a,c) Cathode and b,d) anode.

The structure of CEI and AEI of graphite/Li_1.2_Mn_0.58_Ni_0.08_Co_0.14_O_2_ full‐cell with TLE after extensive cycling was further elucidated by time of flight‐secondary ion mass spectrometer (TOF‐SIMS). **Figure** **3**a displays the depth profiles of secondary‐ion fragments representing the composition of CEI formed on the cycled LRLOs cathode. It shows that the CEI includes inorganic segments of BO^−^, PO_2_^−^, and LiF_2_^−^ which are decomposed by LiDFOB, LiDFP, and LiPF_6_, as well as C_2_HO^−^ dominated organic species generated by solvent decomposition. The distribution profiles indicate that the CEI displays an architecture of inorganic/organic/inorganic‐rich components from inside to outside. From inner to outer layer, the amount of C_2_HO^−^ increases first and then decreases, the amount of BO^−^ remains unchanged first and then decreases, while the amount of PO_2_^−^/LiF_2_^−^ increases first and then maintains. The amount variations of these species in CEI from inner to outer layer are caused by the decomposition sequences of Li salts and solvents, which matches well with HOMO energy levels. In detail, during the charging process, LiDFOB decomposes first and continuously, resulting in a constant amount of BO^−^ in the inner layer. Then, as the voltage increases, the solvent and LiDFP begin to decompose, providing increasing amount of C_2_HO^−^/PO_2_^−^/LiF_2_^−^ in the inner layer. LiPF_6_ finally decomposes to produce constant PO_2_^−^/LiF_2_^−^ in the outer layer. Figure 3b displays the structure the AEI formed on the cycled graphite anode. The AEI presents an architecture of inorganic/organic‐rich components from inside to outside and is distinct from the CEI. Specifically, the outer layer is rich in organic species of C_2_HO^−^ and the inner layer is rich in inorganic products of BO^−^/PO_2_^−^/LiF_2_^−^, indicating the Li salts of LiDFOB/LiDFP/LiPF_6_ decompose prior to the solvents, which is consistent with LUMO energy levels. Figure 3c,d is mapping pictures of secondary‐ion fragments, which reveals that AEI shows much more intense signals of C_2_HO^−^ than that in CEI. On the contrary, the CEI exhibits much more intense signals of PO_2_^−^, BO^−^, and LiF_2_^−^. This also confirms that the outer layer of CEI is rich in inorganic components, while the outer layer of AEI is rich in organic components.

### 2.3 Interface Evolution

**Figure 4:**
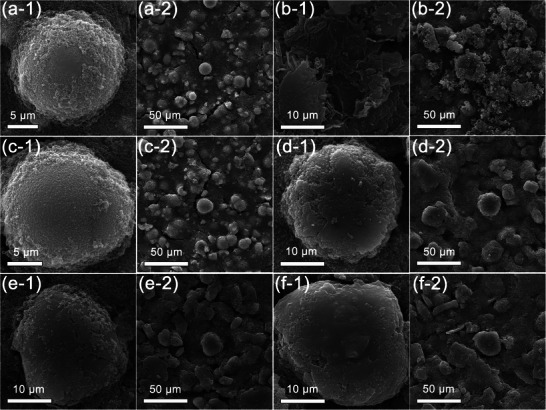
SEM images of a,c) Li_1.2_Mn_0.58_Ni_0.08_Co_0.14_O_2_ cathode and b,d) graphite anode after 100 cycles of graphite/Li_1.2_Mn_0.58_Ni_0.08_Co_0.14_O_2_ full‐cells using different electrolytes: a,b) baseline and c,d) TLE. e,f) SEM images of graphite electrode after 100 cycles of Li/graphite half‐cells using different electrolytes: e) baseline and f) TLE.

The electrode surface morphology may be changed due to the different interface chemistry. To figure out the interface evolution during cycling, scanning electron microscope (SEM) images were applied to investigate the morphology of the both electrode of the graphite/Li_1.2_Mn_0.58_Ni_0.08_Co_0.14_O_2_ full‐cells with baseline and TLE electrolyte after 100 cycles. The pristine state of Li_1.2_Mn_0.58_Ni_0.08_Co_0.14_O_2_ cathode and graphite anode electrodes are shown in Figure S9 in the Supporting Information, and there is a small amount of adhesive and conductive agent on the particle surface, and no obvious interface film is observed. After cycled, the surface of LRLOs cycled in TLE (**Figure** **4**c) is smoother and has less deposits than the sample in baseline (Figure 4a). Figure 4b shows the morphology of graphite electrode cycled in the baseline electrolyte, which presents an obviously uneven deposition layer. More seriously, the graphite structure has been exfoliated and damaged, which might be due to the deposition of transition metal ions dissolved from cathode. In comparison, the graphite morphology cycled in TLE maintains very well, and the interface layer is dense and uniform, as shown in Figure 4d. In order to investigate the crossover effect of LRLOs cathode to the graphite anode, the surface morphology of graphite in Li/graphite half‐cell after 100 cycles was tested as a comparison. As shown in Figure 4e,f, there is no obvious exfoliation of the graphite both in the baseline and TLE electrolyte, and the interface film in TLE is more compact. This comparison suggests that crossover effect of LRLOs cathode to anode in baseline electrolyte destroys the structure of AEI and graphite. However, the AEI rich in F‐, B‐, and P‐containing inorganic components derived from ternary lithium salts is more stable and can resist the cathode‐to‐anode crossover effect.^[^ ^17^, ^18^ ^]^ It can be inferred that the performance improvement of the full‐cell with TLE is benefited by the interface chemistries on the Li_1.2_Mn_0.58_Ni_0.08_Co_0.14_O_2_ cathode and graphite anode.

Electrochemical impedance spectroscopy (EIS) was tested to further investigate the impedance evolutions of the electrode–electrolyte interfaces (Figure S10, Supporting Information). The impedance of a lithium‐ion cell is mainly composed of bulk resistance of the electrolyte (*R*_b_), surface layer resistance at the interface (*R*_sl_), and charge‐transfer resistance (*R*_ct_). This work mainly analyzes the changes of *R*_sl_, which can reflect the evolution of the interface film during cycling. Before cycle, the impedance spectra in different electrolytes are similar. After 100 cycles, the impedance of baseline cell increases more significantly than the TLE cell. It reveals that the continuous interfacial reaction in baseline cell results in the continuous thickening of the interface film, which hinders the transport of Li^+^. In contrast, the impedance of TLE cell is relatively stable, which shows that the F‐, B‐, and P‐rich interface film on both electrodes formed by the preferential decomposition of LiDFOB and LiDFP can inhibit the continuous harmful interface reaction under high voltage or high temperature.

### 2.4 Transition Metal Dissolution and Gas Release

**Figure 5:**
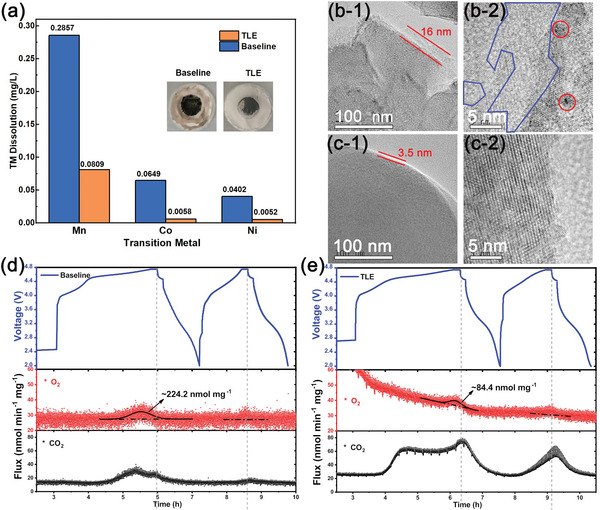
a) Dissolved amount of transition metal on cycled graphite in graphite/Li_1.2_Mn_0.58_Ni_0.08_Co_0.14_O_2_ full‐cells; b,c) TEM images of the CEI layer of cycled Li_1.2_Mn_0.58_Ni_0.08_Co_0.14_O_2_ particles: b) baseline, c) TLE; DEMS measurements of d) baseline and e) TLE of initial two cycles.

In order to explore the crossover effect of cathode to the graphite anode, the dissolution of transition metal (TM) from the cathode is investigated, as shown in the inset of **Figure** **5**a. After 100 cycles in the baseline electrolyte, there are obvious solid residues observed on the separator, while the residues are significantly reduced in TLE. The cycled graphite anodes were tested by inductively coupled plasma optical emission spectroscopy (ICP‐OES) to further determine the amount of transition metal deposition on the surface, as shown in Figure 5a. Obviously, there is severe transition metal (especially Mn) deposition on the graphite electrode in the baseline electrolyte. The deposition amount is as high as 0.2857 mg L^−1^, which is much higher than that in TLE. The results indicate that the inorganic‐rich interphase derived from the TLE electrolyte on the LRLOs cathode can greatly inhibit the dissolution of transition metals and reduce the deposition of transition metals on the anode, preventing the damage to the AEI film. In addition, the inner layer of AEI formed from TLE is also rich in inorganic components, which is highly stable to resist the attack of by‐products.

The interfacial reaction and transition metal dissolution will lead to irreversible phase transitions in the surface structure of cathode materials. Transmission electron microscopy (TEM) and X‐ray diffraction (XRD) tests were performed on the cycled Li_1.2_Mn_0.58_Ni_0.08_Co_0.14_O_2_ cathode. Apparently, as shown in Figure S11 in the Supporting Information and Figure 5b, compared with the pristine Li_1.2_Mn_0.58_Ni_0.08_Co_0.14_O_2_, uneven and thick interface layer (≈16 nm), nanovoids (in the red circle), and disordered lattice/amorphous phase (encircled by blue line) are observed on the surface of baseline sample. The nanovoids and disordered phase are attributed to lattice oxygen release, transition metal ion migration, and transition metal dissolution caused by electrolyte corrosion. The degradation of active material usually starts from the surface and gradually spreads to the inside as the cycling progresses, which is an important reason for the rapid capacity decay. Compared with the sample cycled in baseline, the TEM of cycled LRLOs in TLE displays an even and thin interface layer (≈3.5 nm), and the surface maintains a well‐layered structure, as shown in Figure 5c. XRD was used to identify the crystal structure evolutions of LRLOs after 100 cycles in full‐cells, as shown in Figure S12 in the Supporting Information. For the LRLOs cycled in baseline, the (003) peak and (104) peak shift to lower 2*θ* angle and the peak intensity is significantly reduced after 100 cycles, which are caused by lattice expansion and disordering, while the peak shift and intensity reduction of LRLOs cycled in TLE have been significantly suppressed. The results further demonstrate that the CEI derived from TLE has effectively restrained the structural degradation of LRLOs during long cycling.

The release of lattice oxygen in LRLOs may catalyze the side reactions at the CEI and produce harmful products such as HF and H_2_O, resulting in the transition metals dissolution and irreversible phase transformation. Here, we used differential electrochemical mass spectrometry (DEMS) to detect the gases generation (O_2_ and CO_2_) during the initial two cycles as shown in Figure 5d,e. In the first cycle, when charged above 4.5 V, the oxygen anion participates in redox reaction, and the lattice oxygen on the surface of LRLOs is easily released or even oxidized to O_2_. The oxygen release will deteriorate the structure of LRLOs and cause the reduction of transition metal ions, which lead to severe capacity and voltage decays. Therefore, inhibiting the release of lattice oxygen is an effective method to prevent the capacity and voltage decays. As shown in Figure 5d,e, compared with the baseline cell, O_2_ release in the first cycle of the TLE cell are greatly reduced, suggesting the F‐, B‐, and P‐rich inorganic/organic CEI can effectively inhibit the lattice oxygen release of LRLOs. It is worth mentioning that the TLE cell releases more CO_2_ due to the decomposition of LiDFOB to form inorganic‐rich CEI.

Figure S13a,b in the Supporting Information shows the in situ XRD of Li_1.2_Mn_0.58_Ni_0.08_Co_0.14_O_2_ cycled in baseline and TLE electrolyte during initial two cycles. As shown in Figure S13a (Supporting Information) of the baseline cell, during the first charge at the slope region (2–4.4 V) of the charge profile, the 2*θ* of (003) peak continues to decrease with the Δ*θ*_1_ = 0.143, indicating the interplanar spacing of (003) plane and *c*‐axis are increased due to the increased Coulomb repulsion between the adjacent MO_2_ layers in *R‐3m* phase when Li^+^ is extracted. In the plateau region (4.4–4.7 V), the peak positions of (003) plane are almost unchanged at first, and then increase substantially with Δ*θ*_2_ = 0.405. The abrupt decrease of the (003) interplanar spacing mainly originates from the lattice oxygen release. Compared to the baseline cell, the TLE cell (Figure S13b, Supporting Information) shows a big difference at the end of initial charge. The 2*θ* change of (003) and (104) peaks at the end of charge are only 0.071 and 0.098 in TLE cell, respectively, which matches well with the reduced oxygen release in the TLE cell and reducing the lattice oxygen release effectively suppresses the lattice volume change of LRLOs. Nevertheless, the (003), (101), and (104) interplanar spacing changes of LiNi_0.8_Co_0.1_Mn_0.1_O_2_ in baseline and TLE electrolyte show little difference as shown in Figure S13c,d in the Supporting Information. This is mainly due to the oxygen release of LiNi_0.8_Co_0.1_Mn_0.1_O_2_ is mild relative to the LRLOs. And the phase transition of LiNi_0.8_Co_0.1_Mn_0.1_O_2_ during charge and discharge is the major factor of the lattice volume change rather than the lattice oxygen release. Therefore, for Li_1.2_Mn_0.58_Ni_0.08_Co_0.14_O_2_, both reducing the lattice oxygen release and constructing the robust CEI in TLE are the main reasons for the improvement of the electrochemical performance. While for LiNi_0.8_Co_0.1_Mn_0.1_O_2_, constructing the robust CEI is the main reason.

### 2.5 Extended Applications of TLE in NRLOs and the Synergistic Effect of CEI and AEI

**Figure 6:**
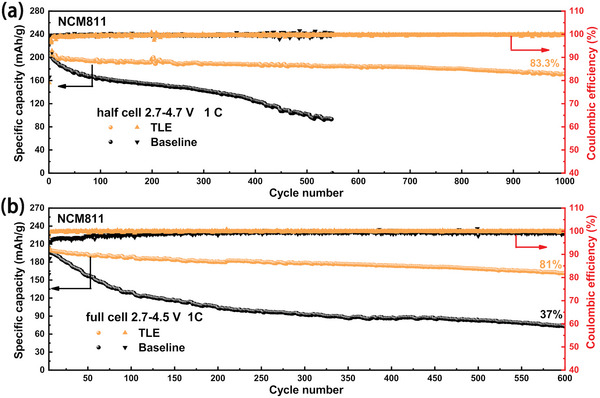
Cycle performance of a) Li/LiNi_0.8_Mn_0.1_Co_0.1_O_2_ half‐cells and b) graphite/LiNi_0.8_Mn_0.1_Co_0.1_O_2_ full‐cells with baseline and TLE electrolyte.

Electrochemical performance of the LiNi_0.8_Mn_0.1_Co_0.1_O_2_ (NCM811) cathode was also evaluated to verify the universal application of TLE in 4.7 V layered oxide cathode cells. **Figure** **6**a shows the cycle performance of the Li/LiNi_0.8_Co_0.1_Mn_0.1_O_2_ half‐cells with baseline and TLE electrolyte in the voltage range of 2.7–4.7 V at 1 C (1 C = 200 mA g^−1^). The capacity retention of the half‐cell with TLE achieves 83.3% after 1000 cycles, which has been greatly improved compared with baseline cell. The capacity retention of the graphite/LiNi_0.8_Co_0.1_Mn_0.1_O_2_ full‐cells with TLE is 81% after 600 cycles, while that of baseline full‐cell is only 37% as shown in Figure 6b. The much higher Coulombic efficiency and better maintained charge and discharge platform of TLE cell (Figure S14, Supporting Information) also indicate that the stable CEI and AEI are also successfully constructed in this cell.

To further investigate the interface stability at higher temperature, the electrochemical performance of the cell with different electrolyte at 45 °C has been compared. As shown in Figure S15a in the Supporting Information, the Li/Li_1.2_Mn_0.58_Ni_0.08_Co_0.14_O_2_ half‐cell with TLE shows much higher capacity retention than the baseline cell after 200 cycles within the voltage range of 2.0–4.7 V at 45 °C. The Li/LiNi_0.8_Co_0.1_Mn_0.1_O_2_ half‐cell with TLE also achieves much higher capacity retention at 45 °C within 2.0–4.5 V (Figure S15c, Supporting Information). Since the side reactions at the electrode–electrolyte interface is accelerated at high temperature, the higher cycle stability demonstrates the interface film with high inorganic content can still ensure the interfacial stability at high temperature and effectively inhibits the interfacial side reactions and the transition metal dissolution at high voltage and high temperature. The cycle performances of Li_1.2_Mn_0.58_Ni_0.08_Co_0.14_O_2_ and LiNi_0.8_Co_0.1_Mn_0.1_O_2_ cathodes at 0 °C were also evaluated (Figure S15b,d, Supporting Information). The cells are activated at 0.1 C and 25 °C for the first cycle, and are subjected to a long cycle at 0.5 C or 1 C under 0 °C. The Li_1.2_Mn_0.58_Ni_0.08_Co_0.14_O_2_ TLE cell exhibits a high‐capacity retention of over 85% after 300 cycles, while the cell with baseline electrolyte experienced rapid capacity decay after 120 cycles. The LiNi_0.8_Co_0.1_Mn_0.1_O_2_ TLE cell delivers capacity retention of 88% after 500 cycles, much higher than that of baseline cell. It suggests the F‐, B‐, P‐rich organic–inorganic composite film provides high Li^+^ diffusion rate through the interface even at low temperature of 0 °C.

**Figure 7:**
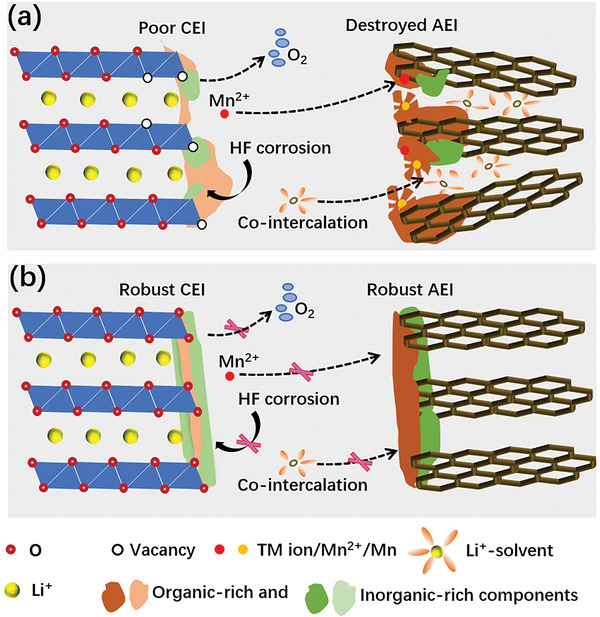
Schematic illustrations of the mechanism of a) baseline‐induced and b) TLE‐induced CEI on layered oxide cathode, AEI on graphite and the interaction between CEI and AEI.

According to the above discussion results, the synergistic effect of CEI and AEI chemistry in the TLE electrolyte can be established. As shown in **Figure** **7**b, the robust inorganic/organic/inorganic‐rich architecture CEI with more F‐, B‐, and P‐rich inorganic components considerably alleviates the parasitic electrolyte oxidation reaction, O_2_ release, and transition metal dissolution, significantly promoting the cycle stability of cathodes under high voltage. The largely reduced amount of transition metal deposition (mainly Mn^2+^) on the anode prevents the chemical destruction of AEI. Meanwhile, the formation of robust inorganic/organic‐rich architecture AEI with more F‐, B‐, and P‐rich inorganic components can effectively protect the anode against the attack of transition metal ions and stabilize the interface, and also can prevent the solvent co‐intercalation that will damage the graphite structure. In sharp contrast, as shown in Figure 7a, the uneven and delicate organic‐rich CEI derived from the baseline electrolyte causes severe interfacial side reaction, O_2_ release, and transition metal dissolution, deteriorating the cycle performance of cathodes. The Mn^2+^ deposits on the anode and catalyzes the dissociation of AEI, which causes solvent co‐intercalation to destroy the graphite structure and further promotes the electrolyte to decompose, leading to the cell failure.

## 3 Conclusion

In summary, this work demonstrates that the LRLOs and NRLOs can be stabilized at a high charge cut‐off voltage of 4.7 V by formulating a TLE. By controlling the frontier molecular orbital energy levels of each component in the electrolyte, the robust inorganic/organic/inorganic‐rich architecture CEI and inorganic/organic‐rich architecture AEI were constructed. Compared with the baseline electrolyte, TLE promotes the formation of F‐, B‐, and P‐rich inorganic compounds both on the cathode and graphite anode, greatly suppresses the parasitic reactions, O_2_ release and transition metals dissolution, and reduces chemical attacks of Mn^2+^ on AEI. By synergistically protecting the cathode and anode interfaces, TLE significantly extends the cycle life of high‐voltage LRLOs and NRLOs cathodes. The Li/Li_1.2_Mn_0.58_Ni_0.08_Co_0.14_O_2_ and Li/LiNi_0.8_Co_0.1_Mn_0.1_O_2_ half‐cells exhibit high‐capacity retention 83.3% after 200 cycles at 2.0–4.7 V and 83.3% after 1000 cycles at 2.7–4.7 V, respectively. The graphite/Li_1.2_Mn_0.58_Ni_0.08_Co_0.14_O_2_ and graphite/LiNi_0.8_Co_0.1_Mn_0.1_O_2_ full‐cells exhibit high‐capacity retention 85.1% after 200 cycles at 2.0–4.65 V and 81% after 600 cycles at 2.7–4.5 V, respectively. Furthermore, TLE also provides high cycle stabilities of high voltage cathodes under higher temperature of 45 °C and lower temperature of 0 °C. Reasonably designing the frontier molecular orbital energy levels of each component in the electrolyte to construct an interface with high inorganic content and high ion mobility is a promising technology with minimum cost to optimize the performance of high‐voltage cathodes and paves the way toward next‐generation high‐energy‐density LIBs.

## 4 Experimental Section

### Material Synthesis

Li_1.2_Mn_0.58_Ni_0.08_Co_0.14_O_2_ was synthesized by calcining the mixture of Mn_0.58_Ni_0.08_Co_0.14_(CO_3_)_0.8_ and Li_2_CO_3_ at 500 °C for 5 h and 825 °C for 12 h in the air. LiNi_0.8_Co_0.1_Mn_0.1_O_2_ was synthesized by calcining the mixture of Ni_0.8_Co_0.1_Mn_0.1_(OH)_2_ and LiOH·H_2_O at 490 °C for 5 h and 770 °C for 12 h in oxygen atmosphere.

### Electrolyte Preparation

The lithium salts in the electrolyte were LiDFOB (99%; from innochem), LiDFP (99%, from ZhengLi Technology Co., Ltd.), and LiPF_6_ (99%+; from Alfa), and the solvent was composed of EC (99.9%; from Alfa) and DEC (99%+, from Alfa). The electrolyte formed by 1.0 m LiPF_6_ dissolved in EC/DEC (1:1 by volume) was marked “baseline,” and the electrolyte formed by dissolving 0.5 m LiDFOB, 0.2 m LiDFP, and 0.3 m LiPF_6_ in EC/DEC (1:1 by volume) was named as “TLE.”

### Electrochemical Measurements

Electrochemical performance was measured in 2032‐type coin cells and pouch cells. The cathodes and graphite anode were prepared by mixing the active materials, super P, and polyvinylidene fluoride in *N*‐methyl‐2‐pyrrolidone at mass ratio of 7:2:1. The above slurry was then coated onto the current collector and dried at 100 °C for 20–24 h under vacuum. The mass loading of cathode was ≈4 mg cm^−2^ and N/P ratio was 1.1–1.2. Lithium metal anode (500 µm thickness in coin cell and 50 µm thickness in pouch cell) was purchased from Guangdong Canrd New Energy Technology Co., Ltd. The dosage of electrolyte was 100 µL per cell, and Celgard 2320 was used as the separator.

The coin cell was assembled by sandwiching the separator between the stainless steel working electrode and lithium metal to measure the cyclic voltammetry curves, at a scan rate of 0.5 mV s^−1^ between 2 and 6.0 V. 2032‐type coin‐type cells and pouch cells with different electrolytes were assembled to evaluate cycle performance. For Li_1.2_Mn_0.58_Ni_0.08_Co_0.14_O_2_, the cells were first preactivated at 0.1 C for one cycle and then kept cycling at 0.5 C (1 C = 250 mA g^−1^), performed at 25 °C between 2.0 and 4.7/4.65 V for half/full‐cell. For LiNi_0.8_Co_0.1_Mn_0.1_O_2_, the cells were first preactivated at 0.2 C for five cycles and then kept cycling at 1 C (1 C = 200 mA g^−1^), performed at 25 °C between 2.7 and 4.7/4.5 V for half/full‐cell. The cycle performance of Li_1.2_Mn_0.58_Ni_0.08_Co_0.14_O_2_ and LiNi_0.8_Co_0.1_Mn_0.1_O_2_ at 0 and 45 °C was also measured in coin‐type cells. EIS was performed on a Princeton P4000 frequency response analyzer in the range of 100 000 to 0.01 Hz.

### Material Characterizations

Surface morphologies were measured with an SEM of TESCAN VEGA3 SBH. XRD was implemented using PANalytical Empyrean diffractometer equipped with Cu K*α* radiation. XPS was used to analyze the surface composition of the cathode and anode, performed on the VG Multi‐Lab 2000 system. Interface film chemical structure was tested using a TOF‐SIMS and the sputtered area was 100 µm × 100 µm, the sputtered speed was 0.33 nm s^−1^. TEM was tested in JEM‐2100 electron microscope. The cycled electrodes were washed with DMC for 5 min, and then dried in a vacuum oven at 105 °C for 10 h before use. For the experiment of the DEMS (Shanghai LingLu Co., Ltd.) tests, coin cell with holes in the cathode was first assembled. And then the coin cell was assembled into the equipment with two ventholes, where one venthole was used to pump in helium with the flow rate of 1 mL min^−1^, another venthole was used to pump out the helium and other gases generated from the charge/discharge process of the coin cell. Specially, O_2_ and CO_2_ release were monitored during the first two cycles with the constant current charging at 0.5 C from 2 to 4.75 V and then constant voltage charging at 4.75 V for 10 min. The transition metal dissolution was measured as following: First, the cycled cells were disassembled and the anode was taken out. After drying, the anode was placed in 6 mL concentrated nitric acid and heated for full digestion. After digestion, deionized water was added to dilute to 60 mL for the test of ICP‐OES (Prodigy Plus). In situ XRD tests were carried out using the cell from Beijing Science Star Technology Co. Ltd. at the current density of 0.13 C.

### Theoretical Calculations

DFT calculation was performed to calculate the HOMO and LUMO energies of molecules using GAUSSIAN 09 software. And the B3LYP with 6–311G + (d, p) was executed to simulate these molecules, in order to obtain the orbitals of them.

## Conflict of Interest

The authors declare no conflict of interest.

## Supporting information

Supporting Information

## Data Availability

Research data are not shared.
