# Supplementary material for: Interface Engineering via Regulating Electrolyte for High‐Voltage Layered Oxide Cathodes‐Based Li‐Ion Batteries
Source: Adv Sci (Weinh). 2023 Feb 19;10(12):2206714. doi: 10.1002/advs.202206714 (PMC10131869; doi:10.1002/advs.202206714)
Supplement: Supplementary file 1 — Supporting Information [file ADVS-10-2206714-s001.pdf]

Supporting information of

Interface Engineering via Regulating Electrolyte for High-Voltage Layered Oxide Cathodes Based Li-ion Batteries

Fangyuan Cheng‡, Jia Xu‡, Peng Wei, Zexiao, Cheng, Mengyi Liao, Shixiong Sun, Yue Xu, Qing Li, Chun Fang*, Yaqing Lin*, Jiantao Han*, and Yunhui Huang

State Key Laboratory of Material Processing and Die & Mould Technology, School of Materials Science and Engineering, Huazhong University of Science and Technology, Wuhan, Hubei 430074, China

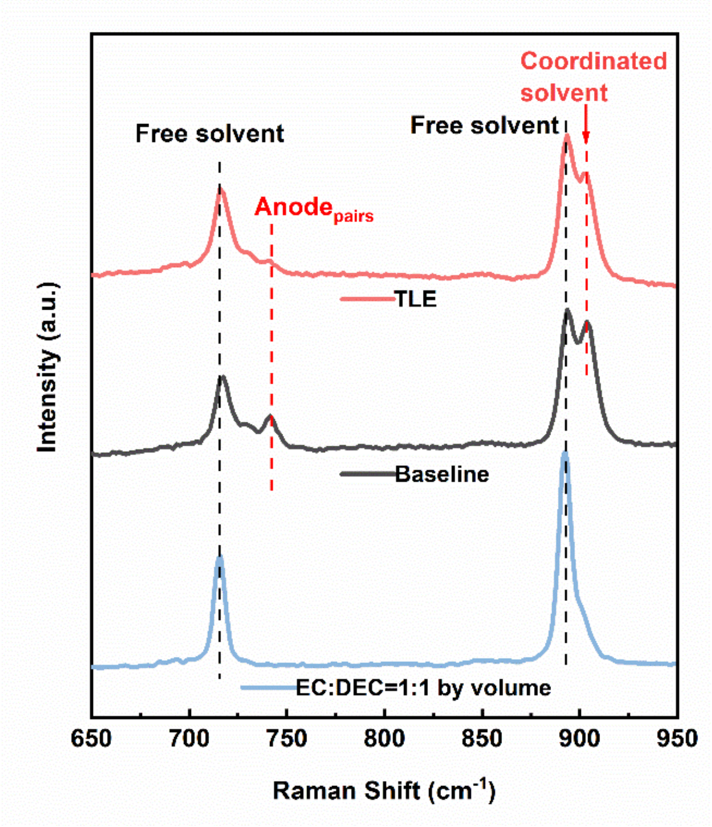

Figure S1. Raman spectra of solvents and electrolytes.

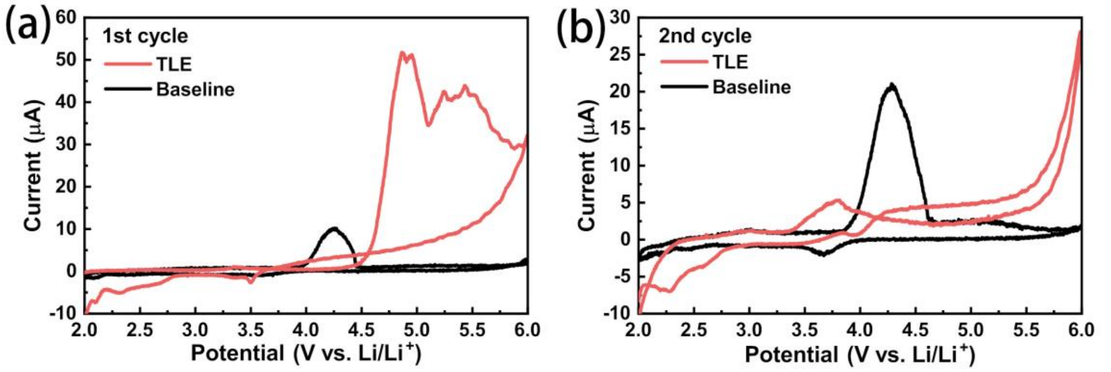

Figure S2. Oxidative potential (Li/stainless-steel) of the baseline and TLE electrolytes at 0.5 mV s^{-1} for (a) 1st cycle, (b) 2nd cycle

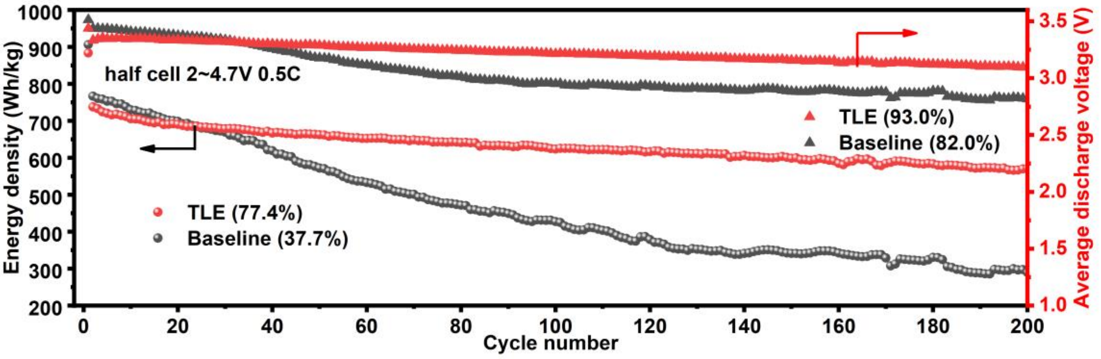

Figure S3. Energy density and average discharge voltage decay of Li/Li1.2Mn0.54Ni0.18Co0.14O2 half cells with baseline and TLE as electrolyte during cycle

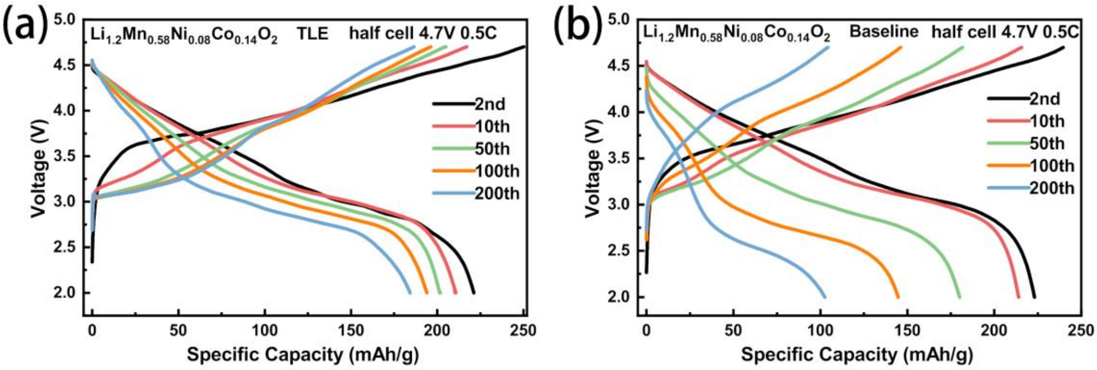

Figure S4. The charge-discharge profiles of Li/Li1.2Mn0.54Ni0.18Co0.14O2 half cells with (a) TLE and (b) baseline as electrolyte

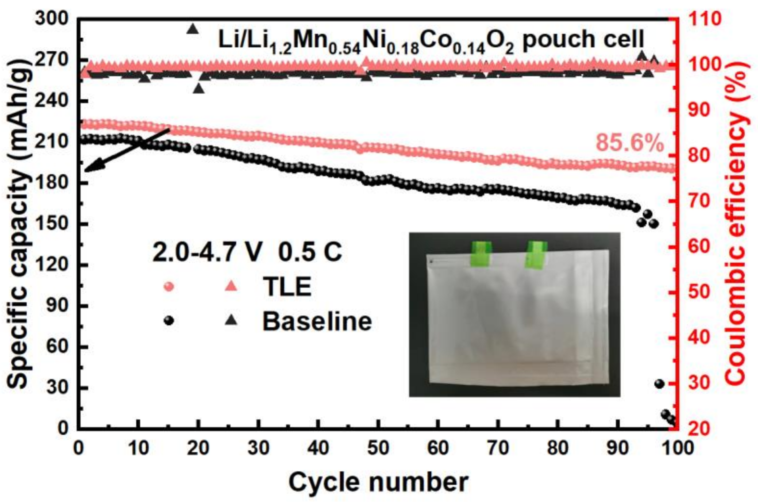

Figure S5. Cycle performance of Li/Li1.2Mn0.58Ni0.08Co0.14O2 pouch cells with baseline and TLE as electrolyte

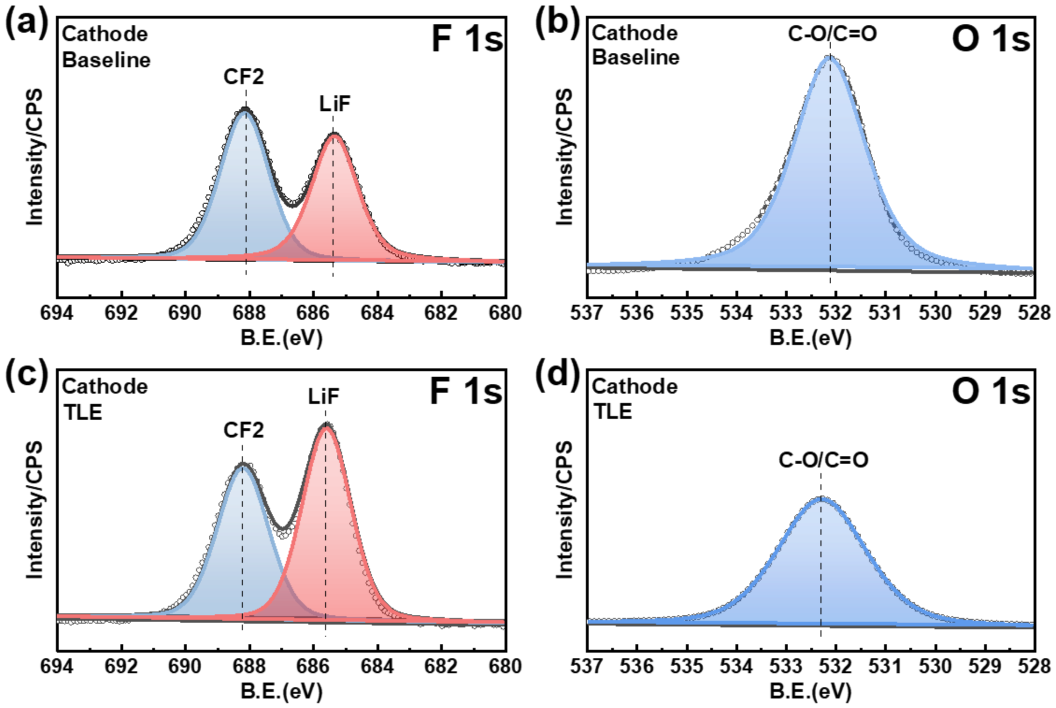

Figure S6. The fitting of F 1s and O1s XPS spectra of CEI layer of graphite/$Li_{1.2}Mn_{0.58}Ni_{0.08}Co_{0.14}O_2$ full cells with TLE and baseline as electrolyte after the 10th cycle

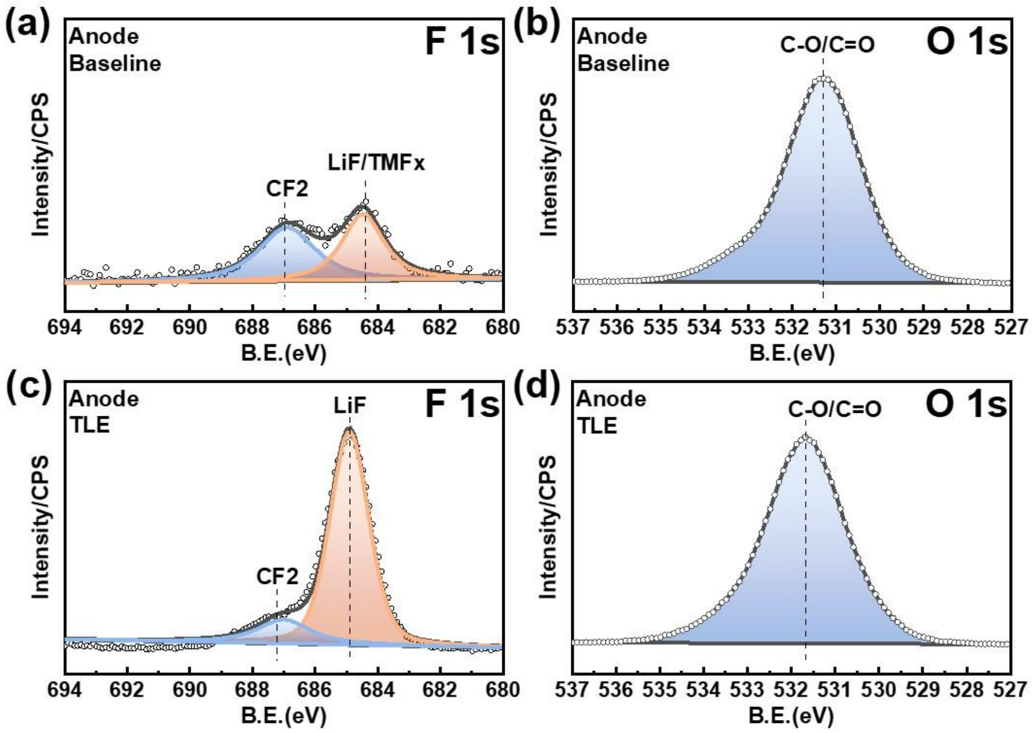

Figure S7. The fitting of F 1s and O1s XPS spectra of AEI layer of graphite/$Li_{1.2}Mn_{0.58}Ni_{0.08}Co_{0.14}O_2$ full cells with TLE and baseline as electrolyte after

10th cycle

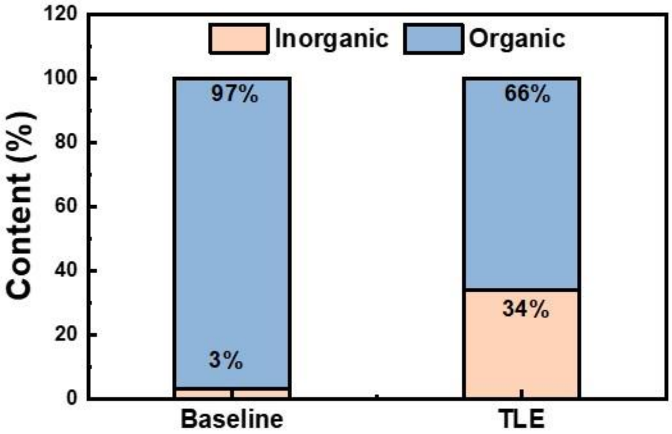

Figure S8. The contents of organic and inorganic components in AEI of graphite/$Li_{1.2}Mn_{0.58}Ni_{0.08}Co_{0.14}O_2$ full cells with TLE and baseline as electrolyte after the 10th cycle

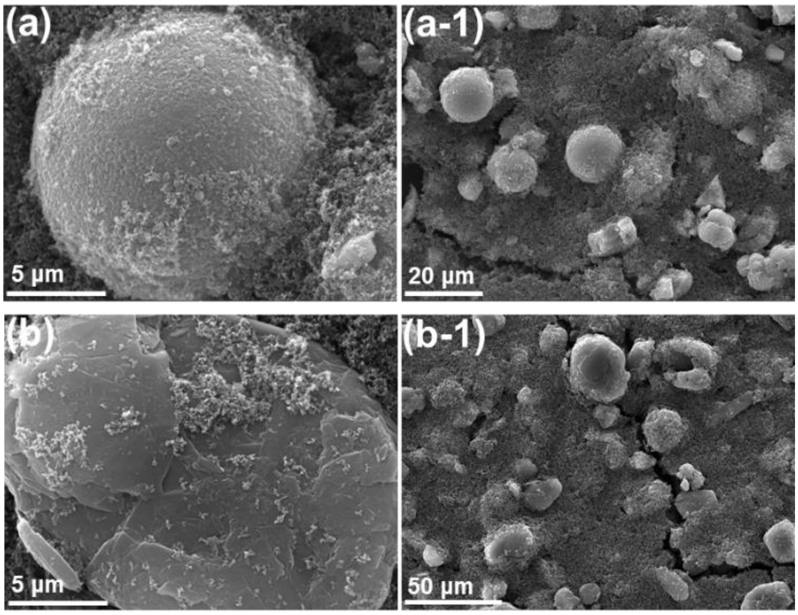

Figure S9. SEM images of the pristine state of (a) $Li_{1.2}Mn_{0.58}Ni_{0.08}Co_{0.14}O_2$ cathode and (b) graphite anode electrodes

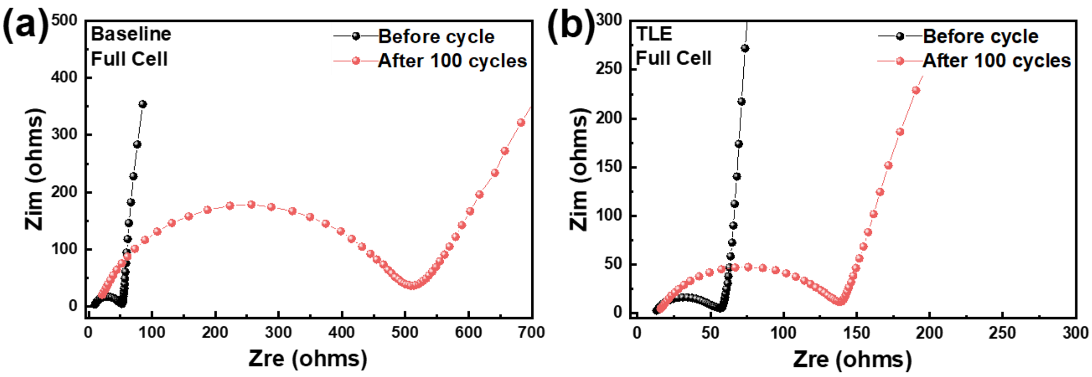

Figure S10. Electrochemical impedance spectra of the graphite/$Li_{1.2}Mn_{0.58}Ni_{0.08}Co_{0.14}O_2$ cells: (a) baseline and (b) TLE.

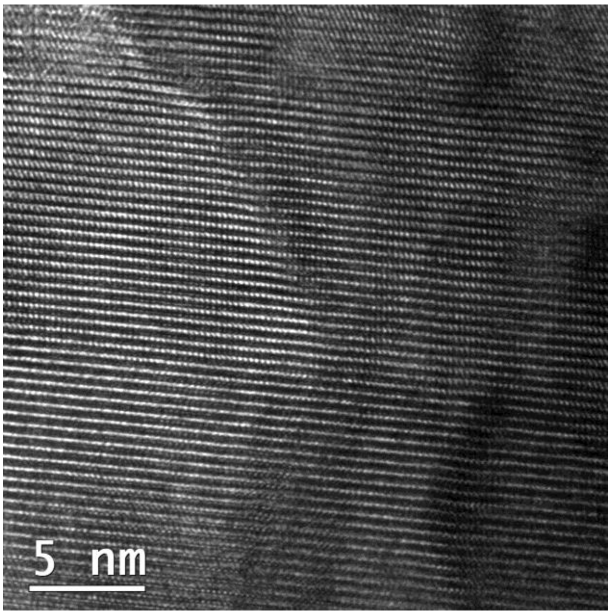

Figure S11. The HRTEM image of pristine $Li_{1.2}Mn_{0.58}Ni_{0.08}Co_{0.14}O_2$

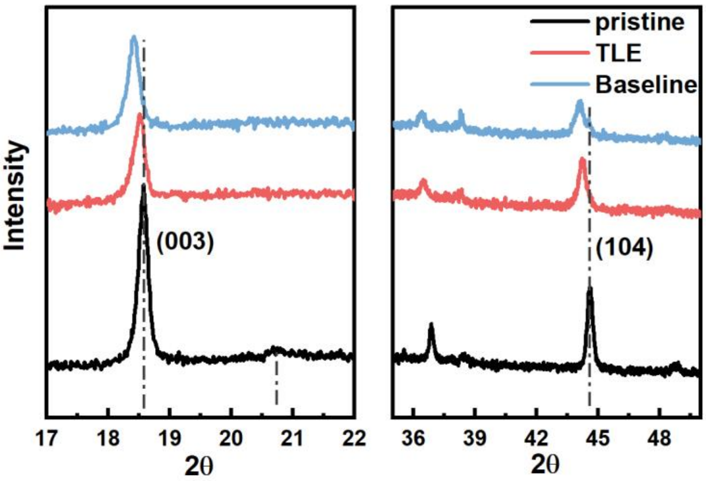

Figure S12. XRD patterns of the $Li_{1.2}Mn_{0.58}Ni_{0.08}Co_{0.14}O_2$ cathode after 100 cycles at 2.0-4.65 V of full cells.

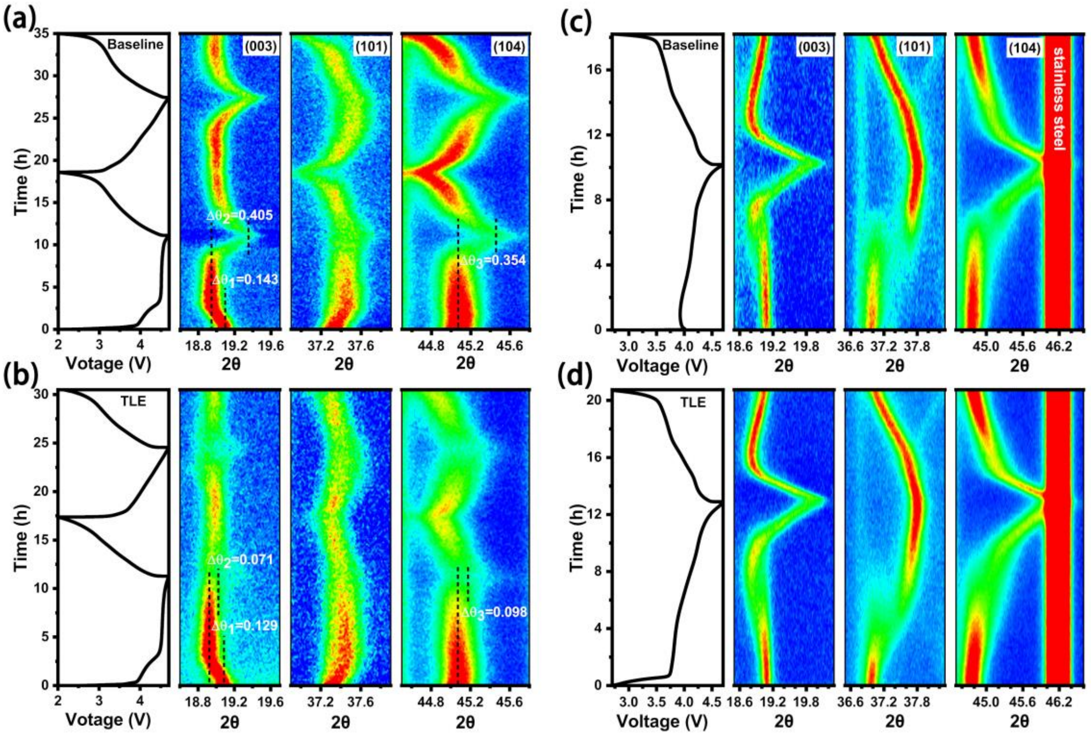

Figure S13. *In-situ* XRD patterns of $Li_{1.2}Mn_{0.58}Ni_{0.08}Co_{0.14}O_2$ with (a) baseline electrolyte and (b) TLE in the initial two cycles, In-situ XRD patterns of $LiNi_{0.8}Co_{0.1}Mn_{0.1}O_2$ with (c) baseline electrolyte and (d) TLE in the first cycle.

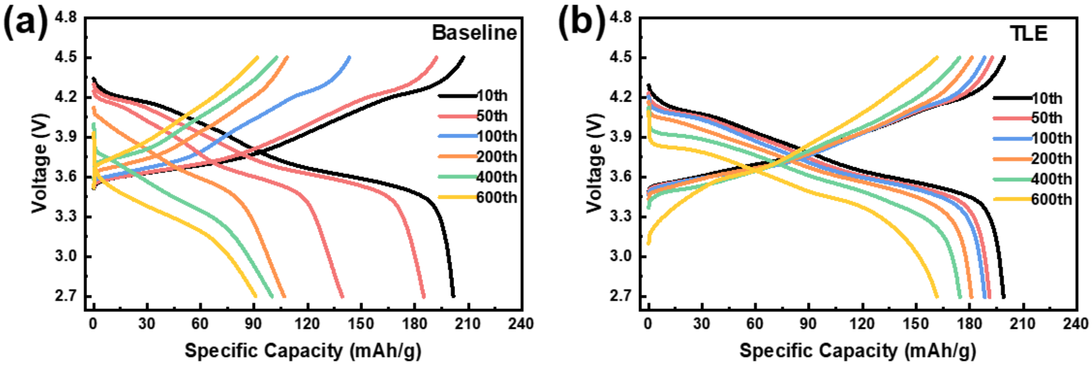

Figure S14. Charge and discharge profiles of graphite/$LiNi_{0.8}Mn_{0.1}Co_{0.1}O_2$ full cells with (a) baseline and (b) TLE electrolytes at different cycle.

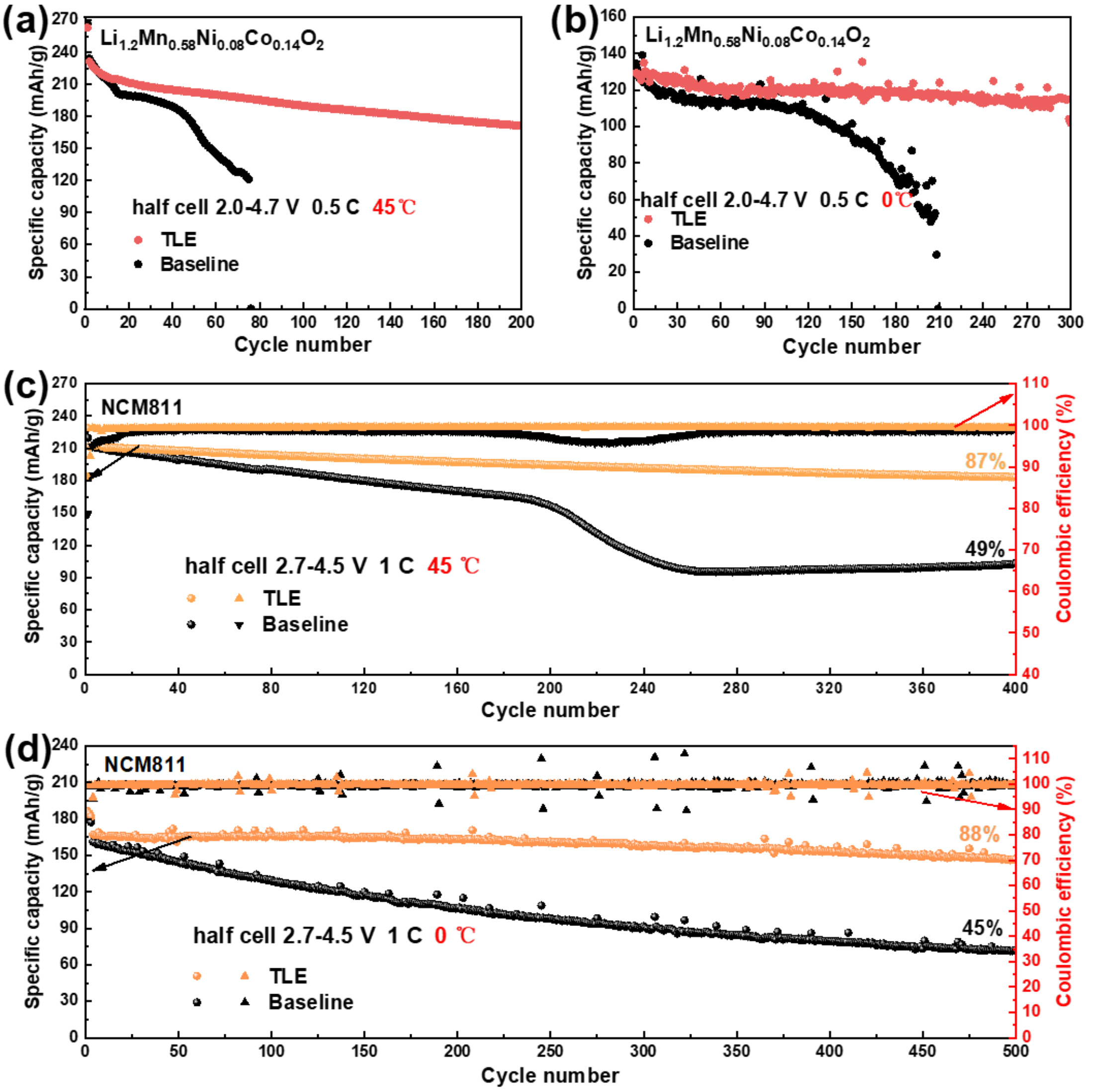

Figure S15. (a-b) Cycle performance of Li/$Li_{1.2}Mn_{0.58}Ni_{0.08}Co_{0.14}O_2$ cells: (a) 45 °C and (b) 0 °C. (c-d) Cycle performance of Li/$LiNi_{0.8}Co_{0.1}Mn_{0.1}O_2$ cells: (c) 45 °C and (d) 0 °C.

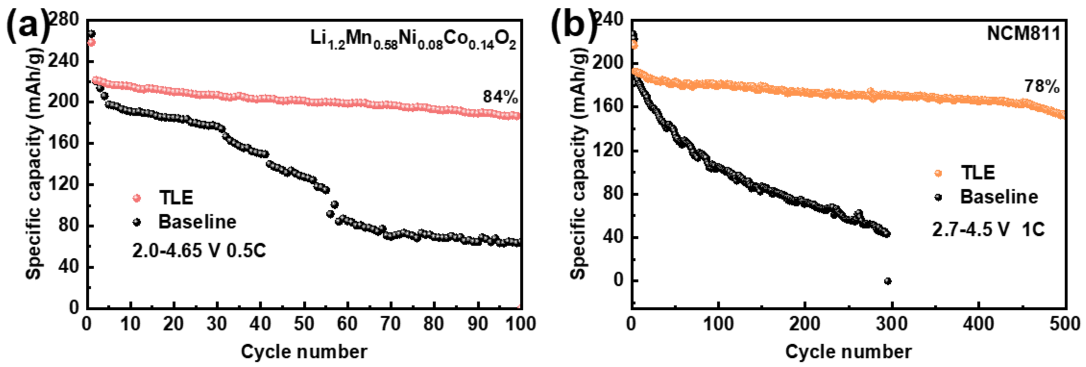

Figure S16. Cycle performance of (a) graphite/$Li_{1.2}Ni_{0.54}Mn_{0.08}Co_{0.14}O_2$ full cells and (b) graphite/$LiNi_{0.8}Mn_{0.1}Co_{0.1}O_2$ full cells with higher mass loading of electrodes (Li-rich: ~7.5 mg/cm^2, NCM811: ~8 mg/cm^2).
